# Therapeutic application of recombinant human ADAMTS-13 improves shock reversal and coagulation status in a trauma hemorrhage and transfusion rat model

**DOI:** 10.1186/s40635-020-00328-w

**Published:** 2020-12-18

**Authors:** Mathijs R. Wirtz, Daan P. van den Brink, Joris J. T. H. Roelofs, J. Carel Goslings, Nicole P. Juffermans

**Affiliations:** 1 Department of Intensive Care Medicine, Amsterdam University Medical Centers, location Academic Medical Center, Amsterdam, The Netherlands; 2 Laboratory of Experimental Intensive Care and Anesthesiology, Amsterdam University Medical Centers, location Academic Medical Center, Amsterdam, The Netherlands; 3 Department of Trauma Surgery, Amsterdam University Medical Centers, location Academic Medical Center, Amsterdam, The Netherlands; 4 Department of Pathology, Amsterdam Cardiovascular Sciences, Amsterdam University Medical Centers, location Academic Medical Center, Amsterdam, The Netherlands; 5 Department of Trauma Surgery, Onze Lieve Vrouwe Gasthuis, Amsterdam, The Netherlands

**Keywords:** ADAMTS-13, Hemorrhage, Trauma, Resuscitation, Coagulopathy

## Abstract

**Introduction:**

In hemorrhaging trauma patients, the endothelium is activated, resulting in excessive endothelial synthesis of von Willebrand Factor (vWF), which may enhance micro-thrombi formation, resulting in obstruction of the microcirculation and endothelial injury, aggravating bleeding, as well as contributing to organ failure. Under normal conditions, vWF is cleaved by the metalloprotease ADAMTS-13. After trauma, ADAMTS-13 levels are reduced.

**Objectives:**

To assess whether recombinant human ADAMTS-13 inhibits endothelial injury and organ failure in a rat trauma-transfusion model.

**Methods:**

Blood products were prepared from syngeneic rat blood according to blood bank standards. Polytrauma was induced in rats by crush injury to the intestines and liver and by fracture of the femur. The rats were hemorrhaged until a mean arterial pressure (MAP) of 40 mmHg was reached. Rats were randomized to receive transfusion of RBCs, FFPs, and platelets in a 1:1:1 ratio to achieve a MAP of 70 mmHg, with or without the addition of ADAMTS-13 (50 μg/kg). Blood samples were assessed for biochemistry and rotational thromboelastometry (ROTEM). Syndecan-1 and VE-cadherin levels were measured as a reflection of endothelial integrity. The amount of leakage of dextran-FITC from the vascular system to the parenchyma in lungs was quantified. To assess inflammation, IL-6 and IL-8 levels were determined. Organ damage was assessed by histopathology.

**Results:**

All rats were severely shocked, with no significant differences in shock parameters between groups. Rats treated with ADAMTS-13 showed signs of a more effective shock reversal (higher blood pressure, lower lactate levels) compared to controls. Also, ROTEM parameters of clot formation in rats receiving ADAMTS-13 improved compared to controls, which was mainly platelet-dependent. Syndecan-1 levels relative to baseline trended to be lower in ADAMTS-13 treated rats compared to controls (107 vs 149%, *p* = 0.08). ADAMTS-13 reduced albuminuria (1.7 vs 4.4 g/L, *p* < 0.01) and organ-specific inflammation (pulmonary IL-6 243 vs 369 pg/mL, *p* = 0.08; splenic IL-6 253 vs 307, *p* = 0.03) compared to controls, but did not improve histopathological scores.

**Conclusions:**

The use of ADAMTS-13 in a rat trauma-transfusion model improves parameters of shock, platelet-driven coagulation, endothelial damage, and organ inflammation. These results suggest that ADAMTS-13 is important in mediating outcome of trauma. Whether ADAMTS-13 can be used as a therapeutic adjunct to treat bleeding trauma patients remains to be determined.

## Introduction

Patients with traumatic bleeding who survive their initial injuries can develop severe complications such as multiple organ failure (MOF). The incidence of post-injury MOF ranges from 15 to 40% with an associated mortality rate of up to 50% [1]. The exact mechanism underlying MOF is unknown, but trauma-induced endothelial dysfunction is thought to play a role [2, 3]. Therapeutic strategies aiming to prevent endothelial dysfunction could therefore limit MOF post trauma.

Endothelial integrity is maintained by the glycocalyx, which is a layer of proteoglycans lining the luminal surface of the vessel wall. When endothelial integrity gets disrupted, glycocalyx constituents such as syndecan-1 and vascular endothelial (VE)-cadherin are shed into the circulation. Elevated levels are detected in traumatically injured patients, suggesting a loss of endothelial integrity [4–6]. Currently, the pathophysiology of endothelial dysfunction in trauma is not fully understood. It was postulated that the ratio of von Willebrand Factor (vWF) to ADAMTS-13 (a desintegrin and metalloprotease with a thrombospondin type 1 motif, member 13) may play an important role [7–9]. After injury, vWF is shed from endothelial cells and mediates platelet adhesion at sites of vascular injury. Monomers of vWF can bind together to form ultra-large von Willebrand factor (ULvWF)-multimers that have the ability to form thrombi spontaneously, due to a very high platelet-binding affinity [10]. The enzyme ADAMTS-13 regulates the size of vWF by cleaving the ULvWF multimers, thereby preventing excess thrombi formation, resulting in a balanced hemostasis.

When increased vWF release outweighs the cleaving capacity of ADAMTS-13, an excess of ULvWF multimers are released in the circulation. An increased ratio of vWF to ADAMTS-13 is found in critically ill septic patients [7] and is consistently associated with organ failure [11], probably due to micro-thrombi occluding the microcirculation and causing endothelial damage.

In trauma patients, a similar pattern to sepsis is found, with low ADAMTS-13 levels [9, 12], whereas vWF is excessively synthesized and shed [8]. In pediatric trauma patients, a dysbalanced ADAMTS-13/vWF antigen ratio was associated with impaired coagulation and increased levels of syndecan-1 compared to healthy controls [12].

Transfusion of plasma products may increase early survival in trauma [13], but this benefit is not related to correction of trauma-induced coagulopathy (TIC) [13–15]. Several preclinical models suggest that plasma can restore the disrupted endothelial barrier function, through restoration of the glycocalyx [16, 17]. Clinical studies in trauma patients support this notion of endothelial improvement after plasma transfusion [18]. In critically ill patients with a coagulopathy, plasma transfusion reduced shedding of vWF and syndecan-1 from the endothelium, which was associated with increased levels of ADAMTS-13 [19]. Given that plasma products contain ADAMTS-13 [20], we postulate that ADAMTS-13 is an important factor in the restoration of endothelial function.

The aim of this study was to investigate the effect of recombinant human ADAMTS-13 on the endothelial integrity, (platelet mediated) coagulation, and MOF in a trauma and transfusion rat model when given as an adjunct therapy to balanced resuscitation. We hypothesized that the administration of ADAMTS-13 leads to less consumption of platelets with concomitant improvement in coagulation and with a reduction in endothelial damage and ensuing MOF.

## Methods

This study was approved by the Animal Care and Use Committee of the Amsterdam University Medical Centers, location Academic Medical Center at the University of Amsterdam, The Netherlands. This study was conducted in compliance with the Animal Welfare Act, the implementing Animal Welfare Regulations, and the Principles of the Guide for the Care and Use of Laboratory Animals.

### Blood product preparation

Preparation of red blood cells (RBC), fresh frozen plasma (FFP), and platelets (PLT) was conducted 2 days before the experiment. For every rat in the experiment, a donor rat was used. Rat blood products were made by collecting blood by heart puncture using a 19G needle and pooled for component preparation, as we have reported before [21].

Blood products were prepared by centrifuging pooled whole blood for 10 min at 1892*g* at 20 °C. Plasma, packed erythrocytes, and the buffy coat were then separated. The buffy coat was diluted with pooled plasma to a hematocrit of approximately 20% and centrifuged for 10 min at 288*g* to separate the majority of the remaining erythrocytes and leucocytes from the platelets. The platelet rich plasma (PLT product) was held under continuous movement, in culture flasks at 22 °C under a 5% CO_2_/95% air mixture, until the day of the experiment [22]. The rest of the plasma was frozen at − 80 °C to serve as FFP products. For the RBC product, saline-adenine-glucose-mannitol (SAGM) was added to the remaining packed erythrocytes to end up with a hematocrit of 55–60% [23], which was stored in 4 °C until the day of the experiment. Blood products were stored according to national Blood Bank standards (Sanquin, Amsterdam, The Netherlands).

At the day of the experiment, FFP products were thawed in ice water for 45–60 min, after which it was kept at room temperature. RBC products were kept at room temperature as well. Just before start of resuscitation, PLT products were removed from the platelet incubator and all blood products were pooled together to achieve a 1:1:1 volume ratio of RBCs, FFPs, and platelets.

### Trauma-transfusion model

Male Sprague Dawley rats were approximately 12 weeks of age, with an average weight of around 350 g. Animals lived in standard cages under normal conditions of a 12:12-h light:dark cycle and received standard lab chow and water ad libitum. Rats were anesthetized using an intraperitoneal injection of KDA (ketamine (1.8 mL 100 mg/mL; Nimatek®, Eurovet Animal Health BV, Bladel, Netherlands), dexmedetomidine (0.5 mL 0.5 mg/mL; Dexdomitor®, Orion Pharma, Espoo, Finland), atropine (0.2 mL 0.5 mg/mL; Pharmachemie BV, Haarlem, Netherlands) and 0.5 mL of NaCl 0.9%), tracheotomized and connected to a mechanical ventilator for the duration of the entire experiment (Babylog 8000, Dräger) [21]. Atropine was administered to prevent respiratory problems due to salivary and excessive bronchial secretions. Maintenance anesthesia consisted of 50 mg/kg ketamine and a pain stimulus was administered every hour to evaluate the level of anesthesia. Body temperature was continuously monitored and controlled by placing the rats on a heated surface set to 37 °C. Polytrauma was induced by performing a midline laparotomy, after which the left and medial liver lobes and 5 cm of the small intestine were crushed using a surgical clamp covered with silicone tubing. Subsequently, the right femur was fractured using a blunt guillotine. By cannulating the carotid artery, the rats were monitored and hemorrhaged until a mean arterial pressure (MAP) of 40 mmHg was reached. After 45 min in a shock state with severe hypotension, rats were transfused at a rate of 8–12 mL/hour in a 1:1:1 ratio of RBC:FFP:PLT through a catheter in the jugular vein. Resuscitation continued until a MAP of 70 mmHg was reached. To objectify the amount of endothelial leakage, rats were infused with a dextran marker of 70 kDa labeled with fluorescein isothiocyanate (0.5 mL 100 mg/mL in NaCl 0.9%; Sigma-Aldrich, St. Louis, USA) 30 min before exsanguination. Six hours after the polytrauma, rats were sacrificed by exsanguination.

### Sacrifice

After exsanguination, the circulation was flushed using heparinized saline (40 μL of 5000 IE/mL per liter saline, Leo Pharma BV, Amsterdam) through the apex of left ventricle and the jugular vein, to remove the excess of intravascular dextran-FITC. After the flushing protocol, the left lung, left kidney, spleen, and medial liver lobe were removed and snap frozen in liquid nitrogen and stored in − 80 °C awaiting fluorescent analysis. The right lung, right kidney, small intestine, and liver were removed and fixed in 10% buffered formalin and embedded in paraffin awaiting further histopathological analysis.

### Randomization

Rats received transfusion of RBCs, FFPs, and platelets in a 1:1:1 ratio to achieve a MAP of 70 mmHg, after which they were randomized to receive a single dose of 50 μg/kg recombinant human (rh) ADAMTS-13 that was prepared as described previously [24] (Sanquin, national Blood Bank, Amsterdam, Netherlands) or the same volume of vehicle (IgG from DAKO, Santa Clara, USA). The dosing of ADAMTS-13 was based on a murine model of intracranial hemorrhage [25], which seemed to be the most effective dose in reducing brain edema. Each group consisted of 8 rats. No sham group was used in this experiment, since previous experiments showed that sham rats do not survive the full duration of the experiment.

### Measurements

#### Biochemistry

At the start of the experiment, blood samples were taken to assess plasma levels of aspartate (ASAT) and alanine transaminase (ALAT), which were measured using standard enzymatic measures. Plasma creatinine levels were measured by colorimetric determination using creatinase. Furthermore, a complete blood count was performed. These measurements were repeated after exsanguination. Arterial blood gases were taken every hour using a blood gas analyzer (Siemens Medical Solutions Diagnostics).

#### Coagulation

The coagulation status was assessed using rotational thromboelastometry (ROTEM, ROTEM Delta, Werfen, Bedford, USA). An EXTEM, INTEM, and FIBTEM assay was performed at baseline, start of resuscitation, during resuscitation, 2 h after start of resuscitation, and at time of exsanguination. After collection of the blood samples, rats received transfusion to compensate the blood loss of the withdrawal.

#### Histopathological examination of organs

For histopathological examination, hematoxylin and eosin (H&E) staining was performed. A pathologist blinded to the treatment groups examined the tissues and graded the severity of injury to the organ on a scale of 0 to 3 (0 = absent, 1 = mild, 2 = moderate, 3 = severe), as we have done before [21]. In short, liver injury was assessed by scoring the presence of necrosis, hemorrhage, portal inflammation, and neutrophil infiltration. Kidney injury was scored on the presence of epithelial necrosis or luminal necrotic debris in the cortical tubules, tubular dilation, neutrophil extravasation, and hemorrhage. Lung injury was assessed by scoring the presence of lung edema, interstitial inflammatory cell infiltration, endothelitis, and hemorrhage. Injury to the small intestine was scored on the presence of diffuse swelling of the villi, neutrophil infiltration in the submucosa, necrosis, and hemorrhage.

Organs were also examined for the presence of thromboembolic events.

#### Immunohistochemical analysis of vascular leakage

To determine the amount of dextran-FITC leakage through the endothelium, pathology slices of the lungs were deparaffinized using alcohol and colored using a rabbit–anti-FITC/anti-rabbit HRP and NovaRed coloring method. Every slice was photographed (using a BX51 with UC90 camera, Olympus, Japan) 5 times using a × 10 magnification, without any vessels in the frame. Images were acquired using a Leica DM-RA Microscope, coupled to a CCD camera (Leica Microsystems, Wetzlar, Germany) equipped with the Image-Pro Plus software (Media Cybernetics, Rockville, USA). Five random inverted pictures were used to set a threshold positive for FITC-70 kDa dextran leakage. Median percentage of area intensity was used as measure for endothelial leakage. All pictures were coded prior to assessments to blind the assessor for treatment allocation.

#### Elisa

All measurements were performed in citrated plasma samples, collected at baseline, and after sacrifice, using enzyme-linked immunosorbent assays (ELISA, all R&D). Levels of vWF antigen were measured. Furthermore, as a reflection of endothelial damage, soluble syndecan-1 and VE-cadherin levels were measured. To assess the amount of organ-specific inflammation, Il-6 and IL-8 levels were determined. Snap frozen organs were lysed in a RIPA buffer (150 mM NaCl, 50 mM Tris-Hcl, 1% nonidet, 0.25% deoxycholate, 0.1% SDS) and centrifuged for 30 min at 5000 rpm at 4 C. The supernatant was collected and Il-6 and IL-8 levels were measured.

### Statistics

Normality was checked by visual inspection of histograms and by Kolmogorov-Smirnov test. Normal values are expressed as mean and standard deviation (SD) and were tested using the student *t* test. In case of no normal distribution, results are presented as median and interquartile range (IQR) and are tested using a Mann Whitney *U* test. In a previous rat model of traumatic brain injury [26], administration of ADAMTS-13 reduced vascular leakage of Evans blue dye, with a mean difference of 3 OD620 (optical density 620 nm) and a standard deviation of 2 OD620. Therefore, to obtain a power of 80%, assuming a 5% significance level and using a two-sided unpaired *t* test, a total of seven rats should be randomized to each group. As we expected a mortality rate within the groups of around 20%, we included eight rats in each group. A *p* value of less than 0.05 was considered to be statistically significant. Statistics were done in IBM SPSS Statistics 24. Graphs were made in GraphPad Prism. ImageJ was used to analyze the amount of vascular leakage in the lungs.

## Results

Not all rats survived the full duration of the experiment. In the control group, 3 rats died prematurely, while in the ADAMTS-13 group, 2 rats died prematurely (cause of death is listed in Supplemental Table 1).

All rats were severely shocked, as reflected by a high base deficit and high levels of lactate. Rats in the ADAMTS-13 group had a higher percentage of their total estimated blood volume bled (see Table 2), but this did not result in significant differences in shock parameters. Compared to the control group, rats in the ADAMTS-13 group had a slightly higher hemoglobin level at baseline; however, this equalized during the shock episode (see Table 1).

**Table 1:** Characteristics at baseline and after trauma but prior to start of resuscitation

	Control		ADAMTS-13	
	Baseline	Hemorrhagic shock	Baseline	Hemorrhagic shock
**Clinical parameters**				
*n*	7		8	
Weight (g)	373.6 (13.4)		360.6 (24.8)	
Temperature (°C)	36.6 (0.8)	36.9 (0.5)	36.8 (0.6)	36.8 (0.9)
**Chemical lab**				
Hemoglobin level (g/dL)	14.0 (1.2)	11.1 (1.0)†	15.7 (0.5)*	11.7 (0.6)†
Creatinine (μmol/L)	25.4 (3.5)	ND	23.4 (2.9)	ND
ASAT (U/L)	63.9 (4.0)	ND	63.3 (9.5)	ND
ALAT (U/L)	54.6 (4.1)	ND	53.1 (6.9)	ND
LDH (U/L)	153.7 (89.0)	ND	129.6 (49.1)	ND
vWF antigen (% of poolplasma)	72 (29–84)	ND	94 (86–101)*	ND
**Blood gas**				
pH	7.44 (0.07)	7.32 (0.11)	7.38 (0.04)	7.33 (0.07)
pCO_2_ (mmHg)	35.4 (5.3)	28.2 (8.7)†	41.0 (7.4)	34.6 (6.6)
HCO_3_^−^ (mEq/L)	23.3 (1.9)	15.1 (6.5)†	23.6 (2.5)	17.5 (1.8)†
BD (mmol/L)	− 0.5 (2.6)	− 9.9 (7.5)†	− 1.4 (1.6)	− 7.6 (2.1)†
Lactate (mmol/L)	0.9 (0.3)	5.2 (4.7)	0.9 (0.3)	3.7 (1.2)†
**Hemodynamics**				
MAP (mmHg)	133 (15)	43 (11)†	149 (9)	44 (3)†
Heartrate (bpm)	282 (32)	265 (54)	270 (18)	252 (31)
**ROTEM**				
Clotting time (s)	49.6 (5.5)	49.3 (3.1)	49.6 (4.6)	43.1 (5.0)†
Mean clot firmness (mm)	69.7 (2.9)	64.0 (4.7)†	69.9 (1.5)	68.9 (2.1)
α-angle (°)	81.7 (1.1)	80.1 (1.8)†	82.5 (0.5)	81.9 (0.6)†
Maximum lysis (%)	5.9 (3.1)	0.9 (0.7)†	2.3 (2.3)*	0.9 (1.5)†

Values are presented as mean and standard deviation (SD) or median and interquartile range (IQR)

*BD* base deficit, *MAP* mean arterial pressure, *ND* not done, *NA* not applicable

*Significantly different from control, *p* < 0.05

†Significantly different from baseline, *p* < 0.05

### Effect of ADAMTS-13 on hemodynamic variables

**Fig. 1:**
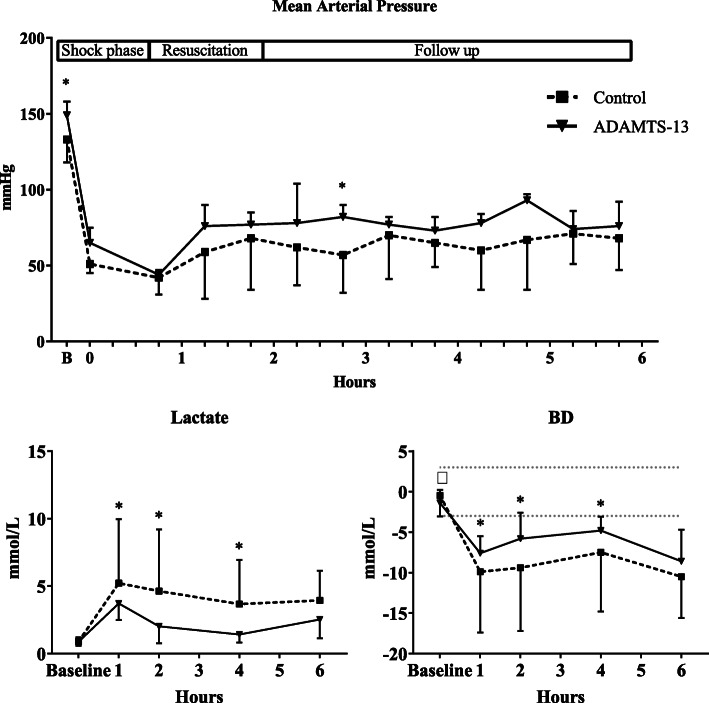
Hemodynamics and biochemistry during the course of the experiment. BD, base deficit. Values presented as mean and standard deviation (SD). *p < 0.05, †*p* < 0.10

Both groups received similar volumes of blood products. Following the ADAMTS-13 treatment, rats maintained a higher MAP throughout the experiment when compared to controls (see Fig. 1). The time to reach the predefined pressure goal was lower in the ADAMTS-13 group compared to controls (10 min, IQR 8–13 vs 34 min, IQR 15–46, *p* = 0.02, see Table 2). In line, lactate levels were significantly lower in the ADAMTS-13 treated rats compared to the control rats (3.7 vs 5.2 mmol/L at *t* = 1, *p* < 0.01). This difference remained until the 4-h timepoint (see Fig. 1). In addition, base deficit was lower in the ADAMTS-13 group compared to control rats, with significant differences at the 1, 2, and 4-h timepoint (see Fig. 1).

**Table 2:** Resuscitation

	Control	ADAMTS-13	***p*** value
Volume bled (mL)	8.0 (1.5)	8.9 (0.9)	0.205
% of estimated total blood volume bled	37.3 (6.7)	43.1 (2.4)	**0.039**
Volume transfused (mL)	9.3 (1.9)	9.5 (0.9)	0.780
Volume per kg transfused (mL)	24.7 (4.9)	26.5 (3.7)	0.780
Time to reach MAP > 70 mmHg (min)	34.0 (14.5–45.5)	9.5 (8.0–12.5)	**0.019**
Median MAP after resuscitation (mmHg)	66 (37–75)	80 (64–83)	**0.032**

Values are presented as mean and standard deviation (SD) or median and interquartile range (IQR)

Estimated total blood volume (mL) = 0.057 x (rat weight in grams)

### Effect of ADAMTS-13 on coagulation parameters

**Fig. 2:**
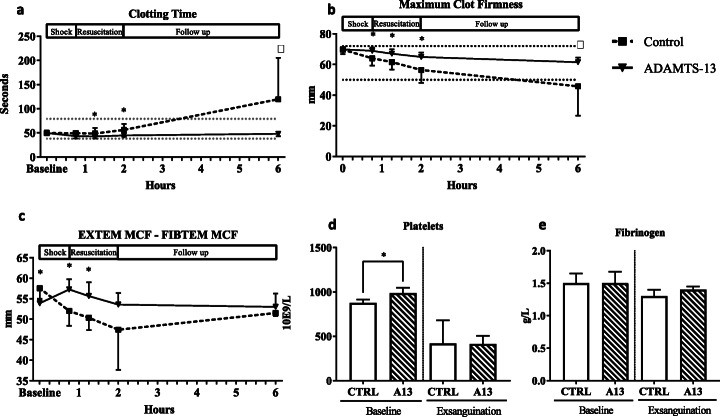
Coagulation parameters. **a** Clotting time measured in EXTEM array over time. **b** Maximum clot firmness measured in EXTEM array over time. **c** Maximum clot firmness of EXTEM and FIBTEM assay subtracted as a reflection of platelet-driven coagulation. **d** Platelet counts at baseline and exsanguination. **e** Fibrinogen levels at baseline and exsanguination. ROTEM values presented as mean and standard deviation (SD). Reference values are according to human standards. Platelet count and fibrinogen levels are presented as median and interquartile ranges (IQR). *p < 0.05

At baseline, platelet counts were lower in the control group compared to the rats receiving ADAMTS-13 (see Fig. 2d), while EXTEM maximum clot firmness (MCF) at baseline was similar between groups. After ADAMTS-13 administration but prior to resuscitation, ADAMTS-13 treated rats showed shorter clotting times and higher MCF compared to controls (see Fig. 2a, b). These differences became more pronounced when resuscitation was initiated, with the biggest differences between groups at the 2-h timepoint.

The contribution of platelet function to clot formation can be assessed by EXTEM MCF subtracted by the FIBTEM MCF value. ADAMTS-13 administration resulted in higher EXTEM–FIBTEM values when compared to controls (see Fig. 2c) until 2 h after administration. FIBTEM levels did not differ between groups (data not shown). Also, fibrinogen levels were not different between groups (see Fig. 2e).

At the end of the experiment, the control group showed a severely decreased MCF, while this parameter, as well as clotting time remained relatively stable in the ADAMTS-13 group. Platelet count was similar between groups at the end of the experiment (see Fig. 2d).

### Effect of ADAMTS-13 on organ injury

**Fig. 3:**
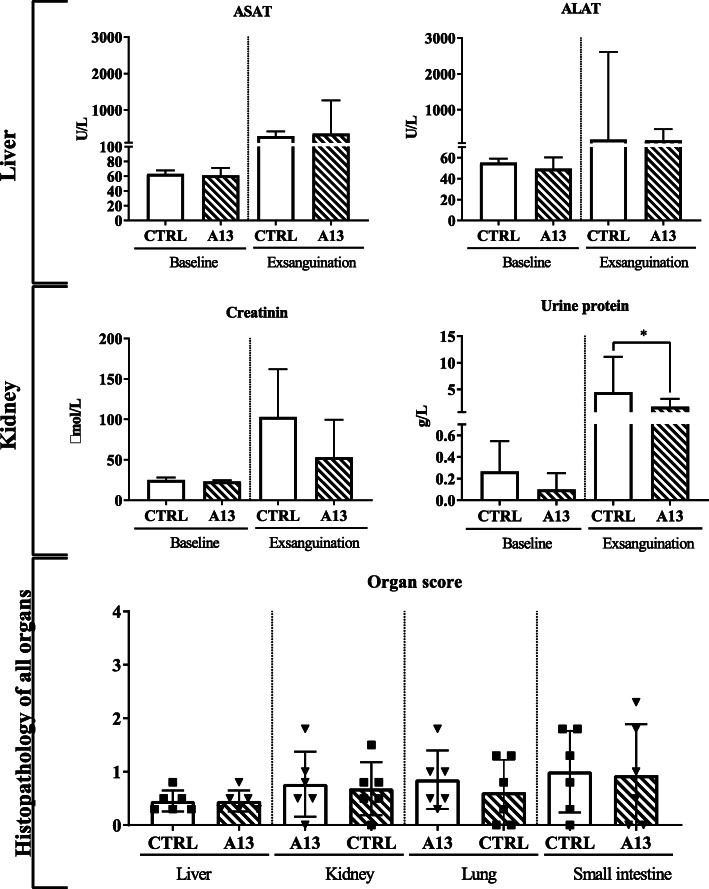
Biochemical and histopathological examination of organ functioning. Levels of aspartate (ASAT) and alanine transaminase (ALAT), creatinine, and lactate dehydrogenase (LDH) were measured in heparinized plasma collected at baseline and exsanguination. Platelet count was measured in whole blood collected in EDTA tubes. Urine was collected at baseline and exsanguination to measure protein levels. Values presented as median and interquartile range (IQR). *p < 0.05

Levels of ASAT, ALAT, creatinine, and urine protein increased over time, suggesting organ injury after shock and transfusion in the rats. Serum creatinine at sacrifice was lower in the ADAMTS-13 group, although not significant (*p* = 0.361). The amount of protein in urine was significantly lower in the ADAMTS-13 group when compared to controls (1.7 vs 4.4 g/L, *p* < 0.01). Levels of ASAT and ALAT did not differ between groups. Histopathological examination of the kidneys and of the other organs did not show any differences between groups (see Fig. 3).

### Effect of ADAMTS-13 on endothelial functioning

**Fig. 4:**
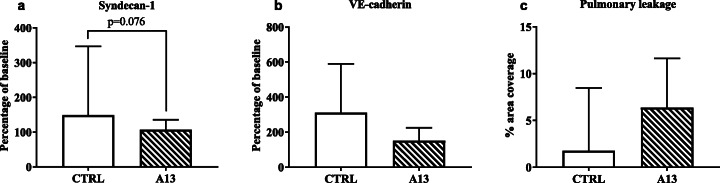
Endothelial functioning. **a** Levels of syndecan-1 after sacrifice relative to baseline values. **b** VE-cadherin after sacrifice relative to baseline values. **c** Percentage of image covered by dextran-FITC as a reflection of endothelial leakage in the lungs. Data is presented as median and IQR

In the group receiving ADAMTS-13, we observed a trend towards lower levels of syndecan-1 relative to baseline (107 vs 149%, *p* = 0.08, see Fig. 4a), suggesting less glycocalyx shedding. We did not find any differences in levels of VE-cadherin relative to baseline (151 vs 312%, *p* = 0.19, see Fig. 4b). Also, the amount of dextran-FITC leakage through the endothelium into the lungs was quantified. This did not differ between the ADAMTS-13 and the control groups (6.4 vs 1.8%, *p* = 0.43, see Fig. 4c).

### Effect of ADAMTS-13 on inflammation

**Fig. 5:**
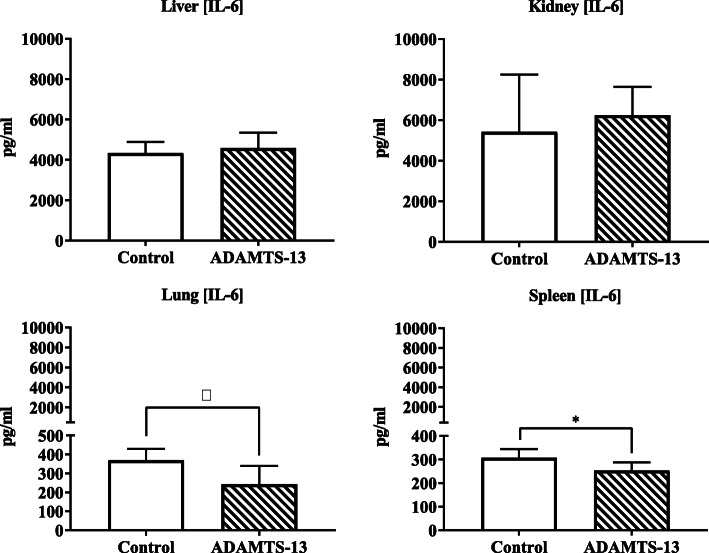
Parameters of organ-specific inflammation. IL-6 levels measured in organ homogenates. Values are presented as median and IQR. *p < 0.05, †*p* < 0.10

Trauma and transfusion resulted in increased amounts of IL-6 levels in all organs. IL-6 levels in kidneys and liver did not differ between the groups. Levels of IL-6 were lower in the spleen (253 vs 307, *p* = 0.03) and trended to be lower in lungs of rats who received ADAMTS-13 (243 vs 369 pg/mL, *p* = 0.08, see Fig. 5). Levels of IL-8 showed a similar trend but were not significantly different between the groups (control vs ADAMTS-13, kidney 2615 vs 2960 pg/mL, *p* = 0.26; liver 1742 vs 2233 pg/mL, *p* = 0.25; spleen 1437 vs 1450 pg/mL, *p* = 0.76; lung 1794 vs 1392 pg/mL, *p* = 0.33).

## Discussion

In this model of trauma-induced coagulopathy and organ failure, ADAMTS-13 given as an adjunct therapy to resuscitation resulted in earlier shock reversal, improved platelet-driven coagulation as assessed by ROTEM, decreased endothelial damage, and partially reduced organ inflammation.

Administration of ADAMTS-13 improved shock reversal in our experimental model, as reflected by a higher MAP, attenuated lactate levels, and a lower base deficit. As we found a trend towards less shedding of glycocalyx constituents into the circulation, this may have been caused by an improved endothelial barrier integrity. In line with this, a protective effect of recombinant ADAMTS-13 on the endothelial barrier function was previously described in a model of traumatic brain injury [26]. However, we could not demonstrate a decrease in vascular leakage of FITC-labeled dextran, nor an improvement in organ failure. This may have been due to a short acting effect of ADAMTS-13, necessitating repeated administration. Alternatively, the model may have lasted too short to demonstrate protection of ADAMTS-13 on the organ level.

Our model is characterized by severely deranged coagulation with prolonged clotting times and a decreased clot strength. We found that ADAMTS-13 improved platelet-driven clot strength as assessed by ROTEM. Improved clot formation most likely was not due to an effect of fibrinogen, as FIBTEM MCF levels throughout the experiment did not differ significantly between groups and fibrinogen levels at exsanguination were also similar. A beneficial effect of ADAMTS-13 on trauma-induced coagulopathy is in line with findings in a model of traumatic brain injury, in which ADAMTS-13 led to reduction in consumption coagulopathy in ADAMTS-13 depleted mice [26]. In our experiment, we found that the effect of the ADAMTS-13 was most clear up to 2 h post-injury. Recombinant human ADAMTS-13 seems to be particularly active in the first 45 min after administration in a single bolus, as was shown in a model investigating therapeutic restoration of ADAMTS-13 levels in rats with neutralizing anti-ADAMTS-13 antibodies [27]. This might explain the fact that the observed improved platelet function in the ADAMTS-13 group did not last until the end timepoint of the experiment. In line with this thought, platelet levels were not different between groups at the end of the experiment. The duration of the experiment may have been too short for ADAMTS-13 to prevent a decline in platelet counts, since this process is time dependent, as was shown in a first-in-human, multicenter dose escalation study in which platelet counts changed after approximately 24 h [28]. Alternatively, it should be noted that platelet counts in rats differ from men, with markedly higher values [29].

Our data indicate that there might be a protective effect of ADAMTS-13 administration on the development of kidney injury, as urine protein levels were significantly lower in this group. Albuminuria is considered to be an early biomarker associated with severe acute kidney injury [30]. However, no difference between groups was found in creatinine levels, nor in the histopathological assessment of the kidneys. Increasing evidence indicates, at least in critically ill septic patients and animal models, that within the first 48 h of acute kidney injury, the defects are mainly functional instead of structural [31], which may explain the biochemical signs of acute kidney injury, but the lack of morphological differences between our groups.

In our experiment, we found lower levels of IL-6 in the lungs and spleen of ADAMTS-13 treated rats compared to controls. This finding was also observed in a mouse TBI model, in which recombinant ADAMTS-13 [25] decreased cerebral inflammation, presumably due to reducing the excess of vWF at the site of injury. This finding was further substantiated by the fact that administration of recombinant vWF upregulated the inflammatory response.

Our experimental model had some limitations. We cannot provide causality of the hypothesis that supplementation of low levels of ADAMTS-13 would result in less thrombi formation with preservation of endothelial integrity. For one, we were not able to reliably measure ADAMTS-13 levels. We attempted to measure ADAMTS-13 levels, but we do not report results as the data did not make sense and we cannot rule out a technical issue with this analysis. Also, micro-thrombi formation is not detectable in histology in this model. Also, the model resulted in dropout of animals, reducing the power. Thereby, some results only showed trends towards a benefit. However, given that platelet-driven coagulopathy improved and the trends all pointed in the same direction, we do feel that our hypothesis may hold ground.

## Conclusion

In conclusion, ADAMTS-13 as an adjunct to resuscitation in our model of trauma, severe shock, and transfusion improves platelet-driven hemostatic efficacy, shock reversal, and reduced inflammation. However, further research in experimental models is necessary before clinical application in trauma patients can be advised.

## Supplementary information

**Additional file 1: Table 1.** Cause of death.

## Data Availability

The datasets used and/or analyzed during the current study are available from the corresponding author on reasonable request.

## Abbreviations

ADAMTS-13: A desintegrin and metalloprotease with a thrombospondin type 1 motif, member 13
vWF: von Willebrand Factor
ULvWF: Ultra-large von Willebrand Factor
MAP: Mean arterial pressure
ROTEM: Rotational thromboelastometry
MOF: Multiple organ failure
TIC: Trauma-induced coagulopathy
RBC: Red blood cells
FFP: Fresh frozen plasma
PLT: Platelets
SAGM: Saline-adenine-glucose-mannitol
KDA: Ketamine, dexmedetomidine, atropine
FITC: Fluorescein isothiocyanate
ASAT: Aspartate transaminase
ALAT: Alanine transaminase
H&E: Hematoxylin and eosin
ELISA: Enzyme-linked immunosorbent assays
IL: Interleukine
SD: Standard deviation
IQR: Interquartile range
