# Supplementary material for: Therapeutic application of recombinant human ADAMTS-13 improves shock reversal and coagulation status in a trauma hemorrhage and transfusion rat model
Source: Intensive Care Med Exp. 2020 Dec 18;8(Suppl 1):42. doi: 10.1186/s40635-020-00328-w (PMC7746419; doi:10.1186/s40635-020-00328-w)
Supplement: Supplementary file 1 — Additional file 1: Table 1. Cause of death. [file 40635_2020_328_MOESM1_ESM.docx]

# Supplemental file

| **Table 1. Cause of death** | | | |
| --- | --- | --- | --- |
| **Group** | **Time of death post-injury** | **Cause of death by macroscopic examination** |  |
| *ADAMTS-13* | 2.5 hours | Intrathoracic bleeding when the carotid artery was cannulated |  |
| *ADAMTS-13* | 5 hours | No cause of death found |  |
| *Control* | Immediately after anesthesia | No cause of death found |  |
| *Control* | 4 hours | No cause of death found |  |
| *Control* | 5 hours | No cause of death found |  |
